# Concentration-driven phase transition and self-assembly in drying droplets of diluting whole blood

**DOI:** 10.1038/s41598-020-76082-6

**Published:** 2020-11-03

**Authors:** Anusuya Pal, Amalesh Gope, John D. Obayemi, Germano S. Iannacchione

**Affiliations:** 1 Order-Disorder Phenomena Laboratory, Department of Physics, Worcester Polytechnic Institute, Worcester, 01609 USA; 2 Department of English, Tezpur University, Tezpur, 784028 India; 3 Department of Mechanical Engineering, Worcester Polytechnic Institute, Worcester, 01609 USA; 4 Department of Biomedical Engineering, Worcester Polytechnic Institute, Worcester, 01609 USA

**Keywords:** Surfaces, interfaces and thin films, Biological physics

## Abstract

Multi-colloidal systems exhibit a variety of structural and functional complexity owing to their ability to interact amongst different components into self-assembled structures. This paper presents experimental confirmations that reveal an interesting sharp phase transition during the drying state and in the dried film as a function of diluting concentrations ranging from 100% (undiluted whole blood) to 12.5% (diluted concentrations). An additional complementary contact angle measurement exhibits a monotonic decrease with a peak as a function of drying. This peak is related to a change in visco-elasticity that decreases with dilution, and disappears at the dilution concentration for the observed phase transition equivalent to 62% (v/v). This unique behavior is clearly commensurate with the optical image statistics and morphological analysis; and it is driven by the decrease in the interactions between various components within this bio-colloid. The implications of these phenomenal systems may address many open-ended questions of complex hierarchical structures.

## Introduction

Phase transition refers to a physical process where a substance experiences a state transformation^1^. The alteration of ice (solid) to water (liquid) and eventually to vapor (gas) due to heat is a typical example of the phase transition. Besides, the appearance of zero electrical resistivity in the superconductivity state, or the transformation of the liquid crystalline into the crystalline phase are just a few other well-studied phase transition examples in the field of condensed matter physics^1,2^. In a similar way, the state of phase transitions in the biological systems equally attracted considerable research attention, where the ideas of statistical mechanics have been applied on many applications, including order parameters, fluctuations, and universality classes. For example, the phase transition of various biomolecules (from a liquid crystalline to gel state) such as lipids, cholesterol, proteins, etc. is found to play an essential role in the selective trafficking and signaling activities across the cell plasma membrane^3,4^. Another vital instance of phase transition includes the sol-gel transformation that modifies the assembling mechanism of the platelets (one of the cellular components of the whole blood) and various fibrin proteins. This particular transition state of the sol-gel is considered to be crucial to healing an injury during the process of blood clotting^5^. Furthermore, the ion-selectivity in nerve fibers alters their structures from the swollen to the shrinking state and influences the process of nerve excitation and conduction. All these biological phase transitioning examples are at the cellular or the molecular level and are driven by both physical and chemical processes^6,7^.

Understanding the drying phases of a multi-component bio-colloidal droplet, such as the whole human blood has attracted many researchers. For example, the spreading, the wetting and the cracking phenomena of the drying droplets of blood (varying in different parameters including substrate, wetting, relative humidity, etc.) have been examined in detail to develop an understanding of the evolving patterns^8–13^. The presence of various cells [red blood cells (RBCs or erythrocytes), white blood cells (WBCs or leukocytes), and platelets (thrombocytes)] make the whole blood a complex bio-colloidal fluid^14^. These cells are suspended in the blood plasma and usually contains water (92% by volume) along with a minute amount of plasma proteins, ions, and hormones in them^15^. The process of drying evolution of the blood droplet starts as soon as a droplet gets deposited on a substrate. The constituent components are dispersed uniformly in the droplet that defines an initial equilibrium state. As the solvent (water) starts evaporating from the droplet, the drying process drives the system out of the equilibrium. The wetting conditions on the substrate enables the evaporation-driven convective flow. Further, water-loss in the droplet concentrates the components facilitating their self-assembling interactions. A signature pattern evolves, and the droplet reaches a new equilibrium state as soon as the drying process is completed.

Recently, diluted blood samples (into different concentration ranges) have shown insightful drying patterns in blood pattern analysis (BPA)^16–19^. Sen et al.^20^, in a different study, examined the effects of diluted blood droplets at a fixed saline concentration by changing the substrate conditions. The study concluded that the transition of the cracking to the non-cracking regime exists for the droplets deposited on the hydrophilic substrate. On the other hand, when the droplet dries on the hydrophobic substrate, the buckling regime is merged to this transition phase. The study primarily focused on the decrease of the RBCs in diluted blood samples, and how the reduction of RBCs affects the mechanical stress developed due to the water loss. Despite the intense research on drying blood droplets, the range of dilution and the use of several experimental techniques (optical microscopy detailing the drying evolution and the final morphology, scanning electron microscopy exploring the deformed blood structures in the dried samples, contact angle measurements giving the idea of the wetting) do not reveal any concentration-driven phase transition region for these diluted blood droplets. In fact, no study is performed on the diluted blood samples to understand the characteristic changes and aim to examine the fundamental understanding of such phase transition and self-assembly^21^ during their drying process and the resulting morphology. The implication of diluted blood samples is crucial and makes this paper different from others’ report. The diluted blood samples lead to changes in the initial equilibrium state that results in reducing the interaction between various components as well as minimizing the biological activities within such multi-colloidal system.

This paper reveals a unique mesoscopic phase transition that is solely driven by the physical process, drying at different concentrations. Exploring the phase transition and the self-assembly patterns in such a multi-component system may reveal much significant information. Keeping these notions in mind, this article aims to address the following vital questions: (1) What are the factors those stimulate a phase transition in the drying droplet of the whole blood? (2) What are the different drying mechanisms leading to the phase transition? (3) Is it possible to explain the complexity of the microscopic structures of the cellular components following the phase transition? If so, to what extent is it possible to do so? And finally, (4) How do the wettability and the interfacial properties of the droplets influence the different environment during the drying process?

Furthermore, the outcome of this research also has the potentiality in applications related to disease diagnosis^22–29^. Several studies have revealed that the resulting final patterns of the drying droplets appear correlated to the initial state^11,12^. Researchers also concluded that patients with chronic kidney disease usually suffer from overhydration or an overload of extracellular water^30^. Moreover, patients in receipt of hemodialysis (a therapy for patients with poorly functioning kidneys) often continue to have low cellular counts after treatments^31,32^. In some cases, this therapy tends to create a low supply of RBCs, which in turn leads to possible anemia cases. Thus, the initial concentration of the whole blood (whether diluted or concentrated) is an important parameter correlated to these pathologies. The conventional method includes the complete blood count (CBC) of almost every patient; however, this alternative drying process proposed in this paper may reflect the (actual) different stages of critical diseases in a more convenient way in the near future. Our proposed method is an initiative to explore their physiological activities.

We attempted to investigate the drying process of the whole blood macroscopically (mm scale) using a bright-field optical microscopy and a contact angle goniometer of the droplet. The statistical image parameters of the microscopy, such as the first-order statistics (mean and standard deviation) are characterized during the drying process^33,34^. The morphological quantities of the final dried state such as the crack width, spacing, etc. are extracted from the optical images at the end of the drying process. Besides, different regions of the morphology of the dried film such as the periphery, the corona (between the periphery and the center), and the central regions are examined in detail ($\upmu$m scale) using a scanning electron microscopy (SEM).

**Figure 1:**
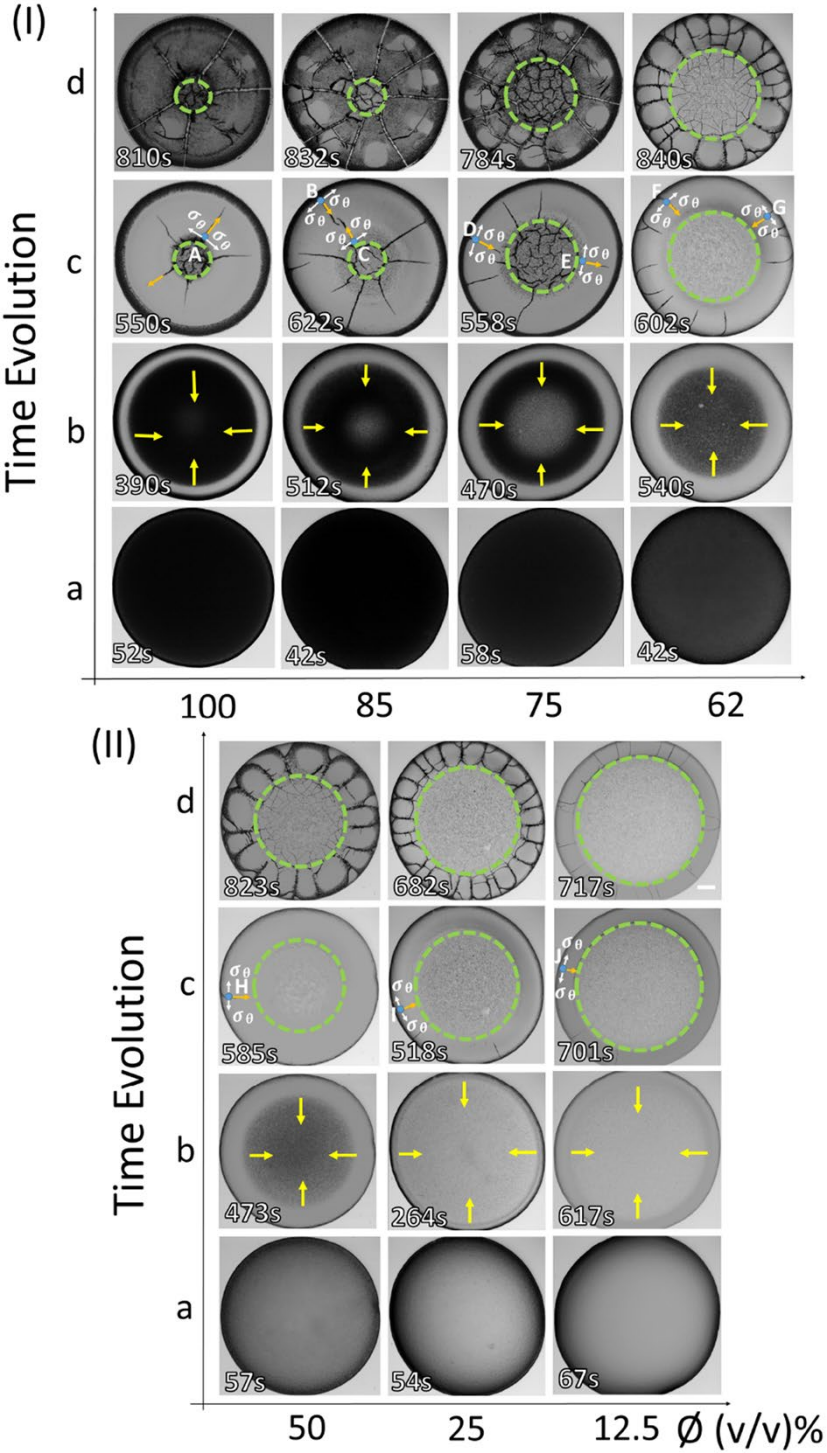
Time-lapse images of the blood droplets with different diluting concentration ($\phi$) are captured during the drying process. These images dictate different stages: (I) from 100 to 62% (v/v), and (II) from 50 to 12.5% (v/v) (in respect to the original volume of the whole blood). The first image was captured at 40–70 s after depositing the droplet on the coverslip. The diluted samples lead to a textural change (from dark to light gray) in the images shown in (**a**). The evolution of the fluid front is indicated with yellow arrows in (**b**). It depicts the second stage of the drying process. This stage occurs at 390–620 s. The appearance of the radial cracks occurs in the third stage that lasts for 500–700 s and is outlined with the orange arrows in (**c**). The respective stress notions ($\sigma _\theta$) are illustrated using white arrows at the A-J points. The morphology of the dried film at the end of the drying process is exhibited in (**d**), where the green dashed circular line separates the corona from the central region at each $\phi$. The scale bar corresponds to 0.2 mm.

**Figure 2:**
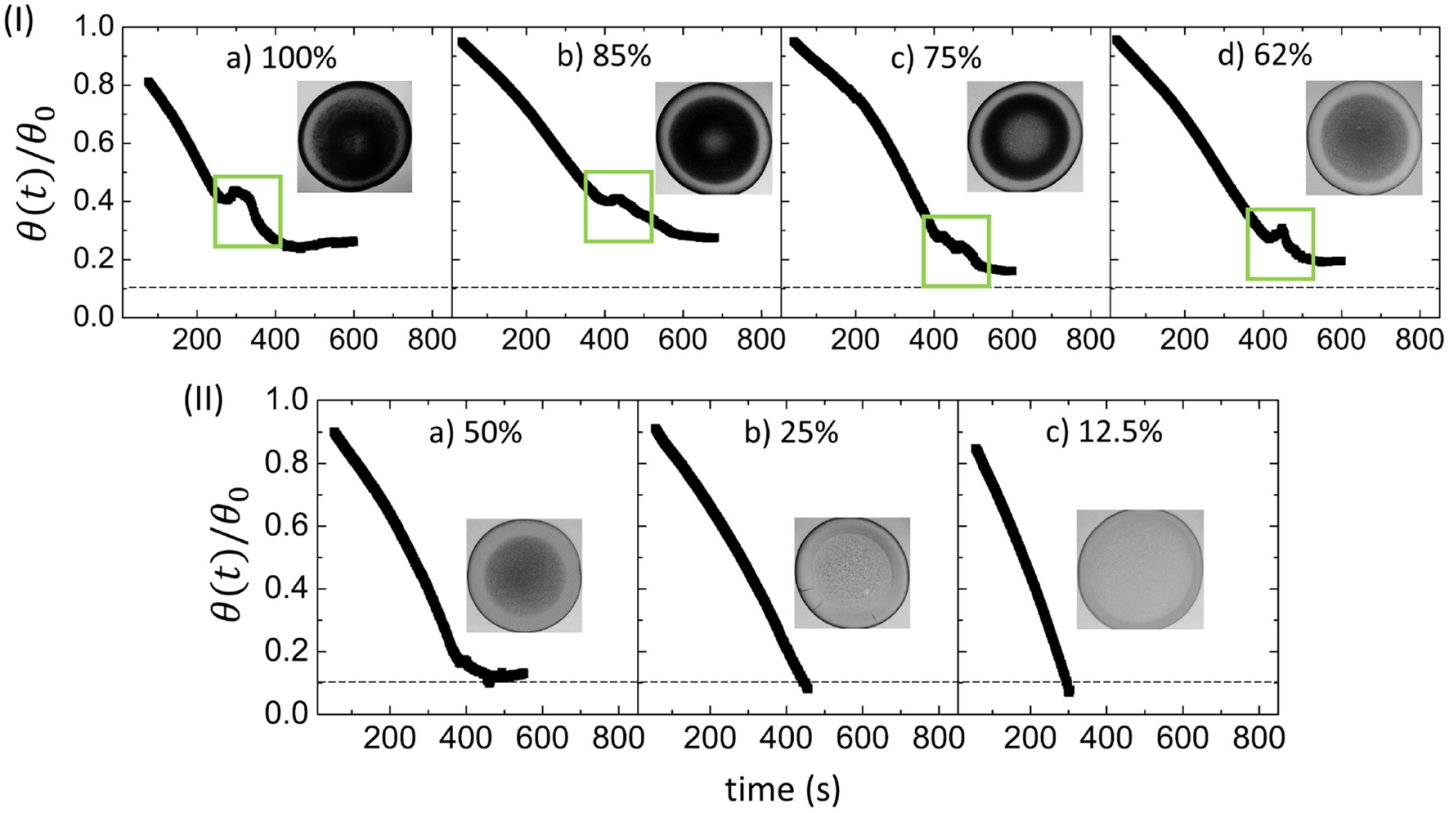
Variations of the normalized contact angle ($\theta (t)$/$\theta _0$) during the drying process of the blood droplets at different $\phi$ ranging (I) from 100 to $62\%$ (v/v), and (II) 50 to $12.5\%$ (v/v) are shown. The normalization of $\theta (t)$ is done by dividing it with the angle at $t=0$ ($\theta _0$). A monotonic decrease in the contact angle measurements is associated with the presence of a peak-like feature (outlined with a green rectangle) is observed in (I). The disappearance of this feature in these measurements is depicted in (II). The macroscopic images are displayed at different time points (in s) points from 100 to 12.5% (v/v) [321 s, 512 s, 470 s, 460 s, 473 s, 518 s, and 617 s respectively for different $\phi$]. The instrumental limit is illustrated with dashed lines.

## Results

### Drying evolution of the blood droplets

Figure 1(I–II) shows the time evolution of the blood droplets diluted at different concentration levels ($\phi$) ranging from 85 to 12.5% (v/v) in respect to the original concentration of the whole blood ($\phi =100\%$). The texture of this first image appears to be dark within the concentration range of 100 to 62% (v/v) (Fig. 1(I)a). However, the dark texture eventually turns out to be gray following the increase of dilution levels (Fig. 1(I–II)a). The physical mechanism of its occurrence is explained in the discussion section. Moreover, a thick peripheral band is observed from 100 to 62% (v/v) (Fig. 1(I)a), which becomes thinner with further dilution from 50 to 12.5% (v/v) (Fig. 1(II)a). Subsequently, a fluid front starts receding from the periphery towards the central region (Fig. 1(I–II)b). As time progresses, the central region becomes devoid of water. The appearance of the light gray texture confirms the evaporation of the water in the central region. This (light gray) texture is prominently observed in samples from 100 to 75% (v/v) dilution; however, it cannot be clearly differentiated in samples from 62 to 12.5% (v/v). This is because the texture of the first image is already light gray at this range of $\phi$, and there is not enough contrast between the two (Fig. 1(II)a,b).

The evaporation of the water and the concentration/assembly of the constituent components promotes the build up of internal stress as the droplet periphery is still pinned to the substrate (coverslip). Figure 1(I–II)c marks the evolution of the cracks (similar to the prior observations discussed in^14,35,36^). The green dashed circular lines separate the corona from the central regions at each $\phi$. The random (small and large) cracks in the central region are first observed from 100 to 75% (v/v). Some of these radial cracks begin propagating towards the periphery, and some towards central regions at $\phi$ of 100 to 62% (v/v) [the points A–E in Fig. 1(I–II)c]. In contrast, their propagation from the periphery towards the central regions is predominantly observed in the diluted samples ranging from 62 to 12.5% (v/v) [the points F–J in Fig. 1(I–II)c]. The radial cracks separate the film into large domains in the corona region as the remaining water evaporates from the droplet during the final drying stage, as shown for $\phi$ from 100 to 75% (v/v) (Fig. 1(I)c). Subsequently, some micro-cracks start originating from these radical cracks in the corona region. However, these micro-cracks cannot propagate in rejoining the dominant cracks in responding to the dominant orthoradial stress field ($\sigma _\theta$). These domains start then begin separating from each other and widening of the radial cracks are observed (Fig. 1(I)d). This process indicated how the film domains detach (delaminate) from the substrate. At this point in the process, the sliding of these films in the radial direction indicates that the energy to adhere to the film is costlier than the sliding energy. This process is predominantly observed from $\phi$ of 100 to $75\%$ (v/v); however, with more dilution, the film adheres more strongly to the substrate. Moreover, the domains which are formed by these radial cracks become narrower with the dilution. The central region of the dried films is found to have the random small crack domains, which are observed for samples from 100 to 50% (v/v). For 25 and $12.5\%$ (v/v), no cracks are detected by the optical microscope (under $5\times$ magnification) in the central region, where these radial cracks are mostly observed in the corona (Fig. 1(I–II)d). A comparison of these dried films textures at each $\phi$ indicates that the whole corona region is of dark gray at $\phi$ of $100\%$ (v/v), whereas, this region is only partially covered by dark gray at $\phi$ of 85 and $75\%$ (v/v). Furthermore, the texture is observed to be of light gray at $\phi$ of 62 to 12.5% (v/v). It should be noted that the optical microscope illumination was nearly constant during the experiments. During this time, the width of the corona region decreases with increasing dilution (decreasing $\phi$). The “coffee-ring” behavior^37^ (similar to what is typically observed in other protein aqueous solutions^38^) becomes evident in samples from $\phi$ of 62 to $12.5\%$ (v/v). V1-V7 in the Supplementary Information show movies of the drying droplet for samples from 100 to 12.5% (v/v), respectively.

Figure 2(I–II) depicts the time evolution of the normalized contact angle of the blood droplets. The normalization of the contact angle ($\theta (t)$) is done in respect to the angle at $t=0$ ($\theta _0$). The $\theta _0$ is determined by a linear extrapolation of the first 200 s of $\theta$ varying with time, where it appears linear. The *y*-intercept provides the normalization in $\theta$. The average slope of this linear extrapolation of the contact angle for all $\phi$ are computed and found to be $-0.14^{\circ }$/s with $\hbox {R}^{2}=0.996$ with very little variation between the samples. The contact angle then shows non-monotonic behavior after 200 s that exhibits a peak marked by a green rectangle in Fig. 2(I)a–d before saturating to a nearly constant with increasing dilution (decreasing $\phi$) from 100 to 62% (v/v). The peak decreases in magnitude and shifts to a later time from 100 to 62% (v/v). No peak is observed with further dilution from 50 to 12.5% (v/v) (Fig. 2(II)a–c). Similar saturation and monotonic behavior are commonly observed in the aqueous protein solutions^38^. The peak is observed from 250 to 400 s at $\phi =100\%$ (v/v) (Fig. 2(I)a); and from 400 to 500 s for $\phi$ varying from 85 to 62% (v/v) (Fig. 2(I)b–d) (denoted with vertical dashed lines). The corresponding macroscopic images during the emergence of this peak are displayed in Fig. 2(I)a–d. The images indicate that the peak originates during the appearance of the fluid front or the gray texture from the periphery and it continues to evolve when the texture starts growing in the central region. In contrast, this peak is not observed for samples from 50 to 12.5% (v/v). The macroscopic images in Fig. 2(II)a–c are captured once the contact angle advances to steadiness. It is to note that the contact angle becomes constant when it is ${\sim } 25\%$ for $\phi$ of 100 and $85\%$ (v/v), ${\sim  } 15\%$ for $\phi$ of 75 and $62\%$ (v/v), ${\sim } 10\%$ for $\phi$ of $50\%$ (v/v), and $<{\sim } 10\%$ of the total value for $\phi$ of 25 and $12.5\%$ (v/v). This suggests that the thickness of the film at the end of the drying process decreases with the diluting concentration ($\phi$).

To validate the peak’s appearance is only specific to the blood samples diluted with de-ionized water, the physiological and environmental conditions were varied to measure the contact angles during the drying process. Different sample batches, and substrate types were taken into account. The diluent was also varied from the de-ionized water to the phosphate buffer saline (PBS) to observe any other possible patterns. The results show that these peaks are present in the diluted blood samples ranging from 100 and $62\%$ (v/v) (Figs. S1-S3 in the supplementary section) for these different setups. However, it is assumed that the nature (shape, size, and time) of these peaks are affected due to these different conditions.

**Figure 3:**
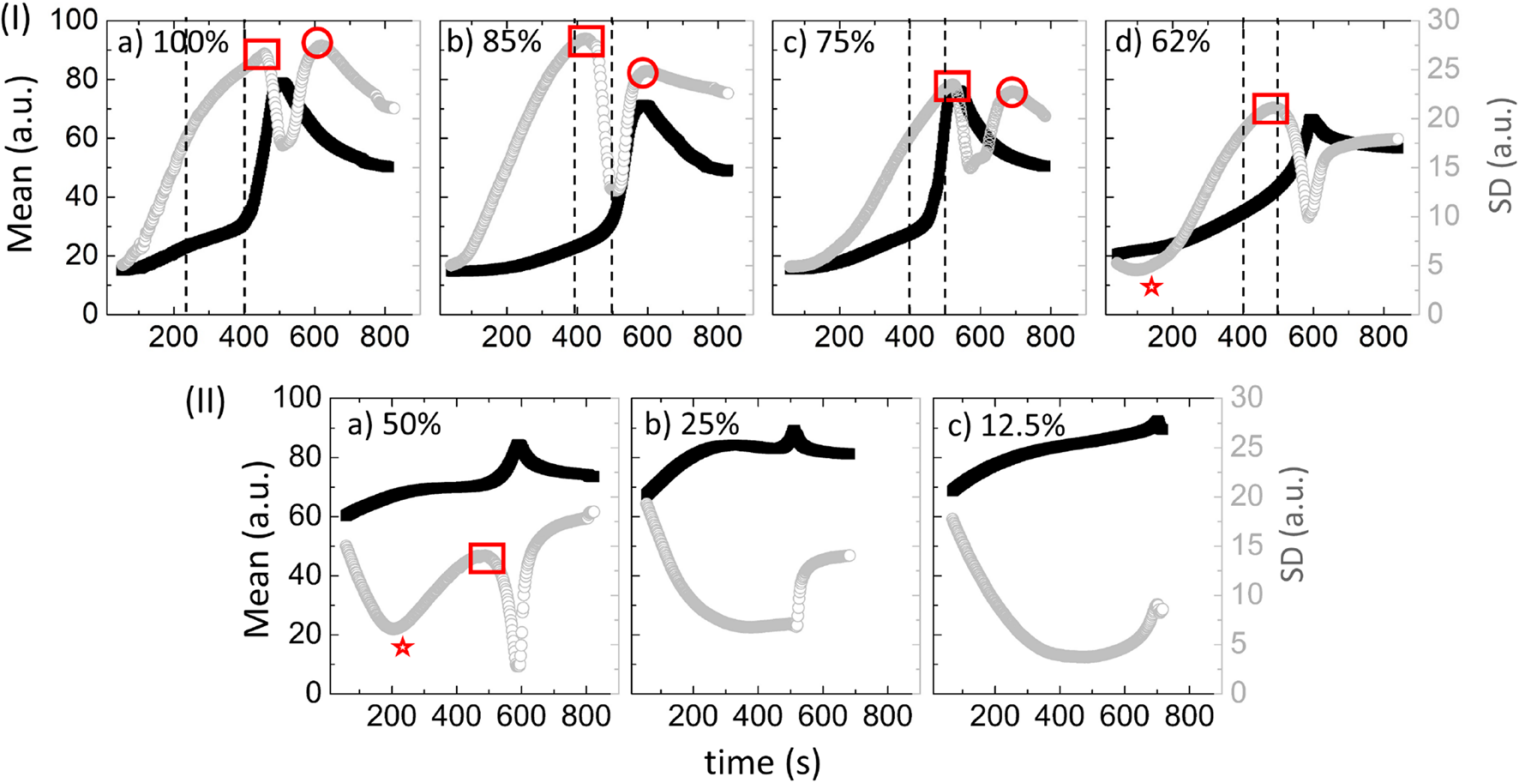
Statistical image analysis of the blood droplets at different $\phi$ from 100 to 12.5% (v/v) during the drying process. (I) and (II) show the drying evolution of the first order statistics (FOS) parameters (the mean on the left *y*-axis and the standard deviation (SD) on the right *y*-axis of the graph). The dashed lines in (I) display the time duration of the presence of the peak-like feature in the contact angle measurements from $\phi$ of 100 to 62% (v/v). The red rectangle and the red circle illustrate the first and the second peaks respectively in the SD. The star introduces the initial dip in the SD values observed from $62\%$ (v/v) onwards.

**Figure 4:**
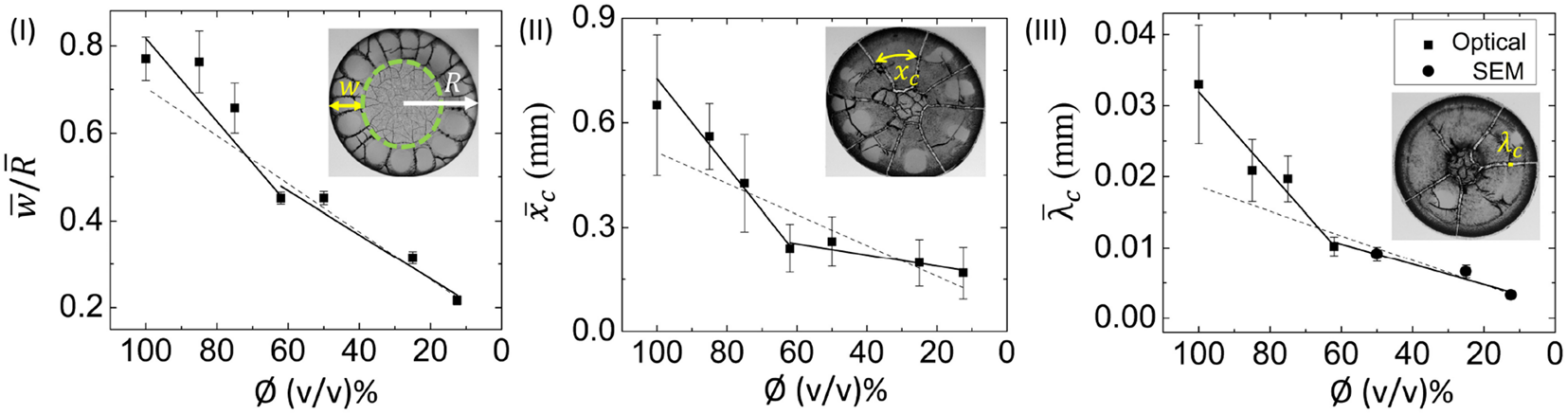
Variation of the averaged corona width ($\overline{w}$ measured in mm) is normalized to the averaged radius of the droplet ($\overline{R}$ measured in mm). It is plotted in (I), the averaged crack spacing ($\overline{x}_c$) is depicted in (II), and the averaged crack width (${\overline{\lambda }}_c$) is exhibited in (III) at different diluting concentrations ($\phi$). The dashed line shows the linear fit for the whole range of $\phi$ from 100 to $12.5\%$ (v/v). The solid lines display two linear fits—first one for the range from 100 to $62\%$ (v/v); and the second one for 62 to $12.5\%$ (v/v). The error bars correspond to the standard deviation. The macroscopic images illustrate the width of the corona (*w*), the radius of the droplet (*R*), the distance between the consecutive radial cracks ($x_c$), and the width between the consecutive radial cracks ($\lambda _c$). The dashed green circular line separates the corona from the central region in the droplet.

Figure 3(I–II) illustrates the parameters of the first-order statistics (FOS). The left *y*-axis of the graph shows the mean intensity ($\frac{1}{N}\sum _{i,j=0}^{N} I_{ij}$) and its right *y*-axis exhibits the standard deviation ($\sqrt{\frac{\sum _{i,j=0}^{N} (I_{ij}-\mu )^{2}}{N-1}}$), where, (*i*, *j*), $I_{ij}$, and *N* are the gray values in a matrix, the pixel matrix of an image, and the number of pixels in the image, respectively. These parameters are extracted from the captured time-lapse images of the droplets at different $\phi$ during the drying process. The dashed lines of Fig. 3(I–II) denote the time range of the peak-like feature in the contact angle measurements. Note that the mean intensity shown in Fig. 3(I–II) depends on the average pixel distribution of the images. These data display three distinct phases—a slow increase, a rapid rise, and a saturation from $\phi$ of 100 to $75\%$ (v/v). The first and second of these three phases; however, could not be distinguished in the 62 to $12.5\%$ (v/v) samples. The large area under the peak is observed in the mean intensity at about 600 seconds for $100\%$ sample, which continuously decreases with increasing dilution, nearly disappearing in the $12.5\%$ (v/v) sample. The SD measurement [gray circles in Fig. 3(I–II)] depends on the global heterogeneity of the images, and is a measure of the image complexity. It exhibits an initial dip (outlined by a red-colored star) from $62\%$ (v/v) onwards. The behavior of these FOS (the mean intensity and especially the SD) parameters changes abruptly at about $62\%$ (v/v) dilution.

### Morphology of the dried blood film

Figure 4(I) illustrates the variation of the averaged corona width ($\overline{w}$) that is normalized to the averaged radius of the droplet ($\overline{R}$). The macroscopic image in Fig. 4(I) illustrates the width of the corona (*w*) and the radius of the droplet (*R*). The increased dilution [$\phi$ from 100 to 12.5% (v/v)] systematically decreases the corona width. The $\overline{w}/ \overline{R}$ is plotted for the whole range of $\phi$, i.e., from 100 to 12.5% (v/v). The slope value (*m*) is found to be $0.0055\pm 0.0006$ $\phi ^{-1}$ with $\hbox {R}^{2}=0.931$. A first linear curve is fixed from 100 to 62% (v/v). Subsequently, a second curve is fitted from 62 to $12.5\%$ (v/v). The slope value of the first linear fit ($m_{1}$) is found to be 0.0095 ± 0.0019 $\phi ^{-1}$, with $\hbox {R}^{2}=0.894$. In contrast, the slope value of the second linear fit ($m_{2}$) is 0.0051 ± 0.0008 $\phi ^{-1}$, with $\hbox {R}^{2}=0.928$. The variation of the averaged crack spacing ($\overline{x}_c$) with $\phi$ is shown in Fig. 4(II). The distance between the consecutive radial cracks ($x_c$) is illustrated in the macroscopic image. The parameter, *m* [the slope value of the linear fit for the range of 100 to $12.5\%$ (v/v)], $m_1$ [the slope value of the linear fit for the range of 100 to $62\%$ (v/v)], and $m_2$ [the slope value of the linear fit for the range of 62 to $12.5\%$ (v/v)] are found to be $0.0045~ \pm ~0.0012$ mm $\phi ^{-1}$ with $\hbox {R}^{2}=0.668$, $0.013~ \pm ~0.0014$ mm $\phi ^{-1}$ with $\hbox {R}^{2}=0.965$, and $0.0016~\pm ~0.0005$ mm $\phi ^{-1}$ with $\hbox {R}^{2}=0.736$ respectively. Fig. 4(III) displays the averaged crack width (${\overline{\lambda }}_c$) variations of each $\phi$. The width between the consecutive radial cracks ($\lambda _c$) is exhibited in the macroscopic image. The optical microscopy at $5\times$ magnification has the resolution to measure the ${\overline{\lambda }}_c$ down to the $62\%$ (v/v) sample. For the ${\overline{\lambda }}_c$, the $m_1$ is extracted as 0.00057 ± 0.00008 mm $\phi ^{-1}$ (with $\hbox {R}^{2}=0.938$). To overcome the limitations of the optical microscopy, the SEM images are used to calculate $\lambda _c$ for $\phi$ from 50 to 12.5% (v/v), where $m_2$ at this range yields 0.00014 ± 0.00002 mm $\phi ^{-1}$ (with $\hbox {R}^{2}=0.920$). Finally, over the whole range of $\phi$, the slope (*m*) for ${\overline{\lambda }}_c$ is 0.00017 ± 0.00003 mm $\phi ^{-1}$ (with $\hbox {R}^{2}=0.835$).

**Figure 5:**
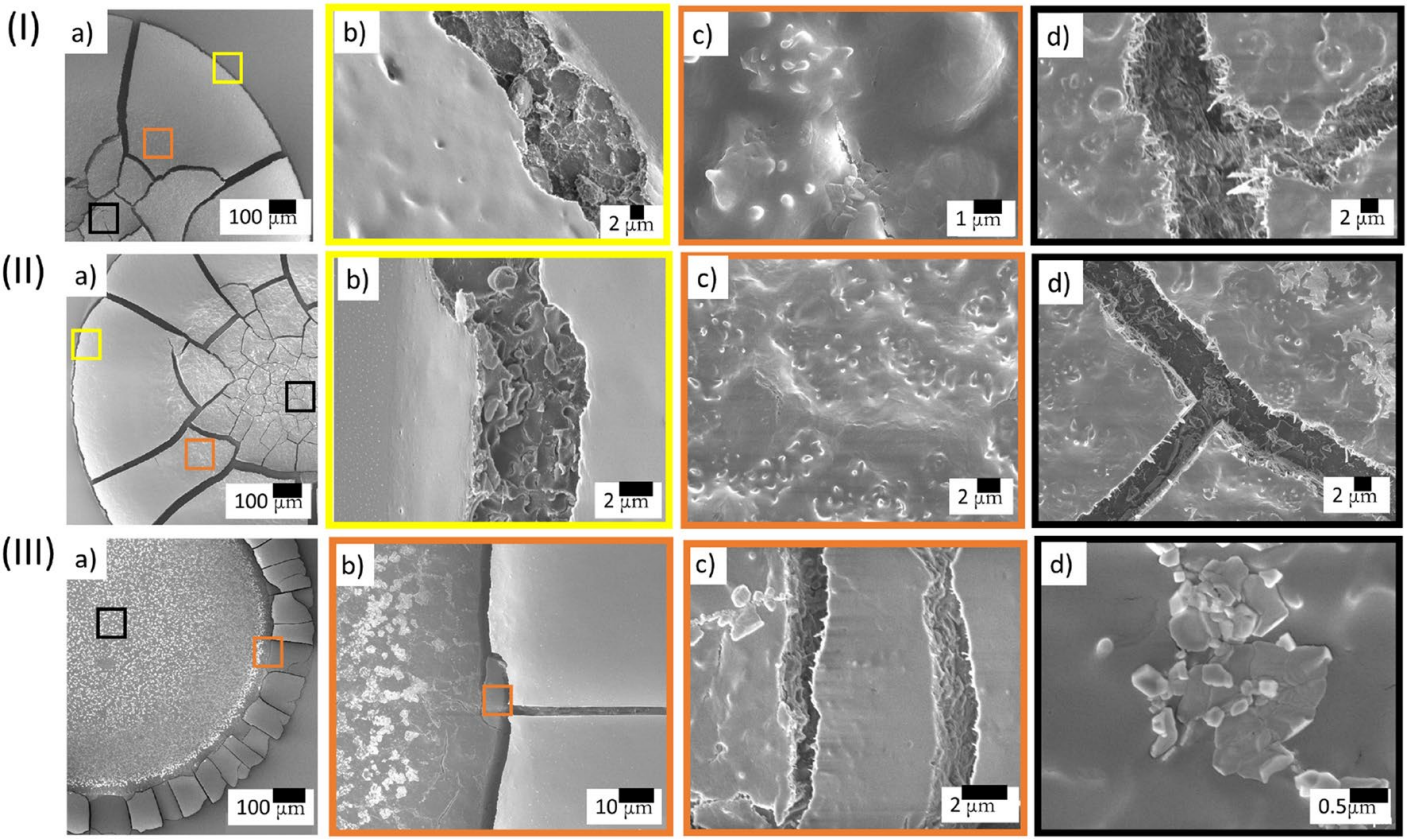
SEM images illustrating the microscopic structures of dried blood droplet at different length scales. The images at $\phi$ of 100, 75 and $12.5\%$ (v/v) are, respectively, depicted in (I–III). The yellow, orange and black squares represent the blood structures at the periphery and corona, the transition of the corona and central regions, and the central region, respectively.

**Figure 6:**
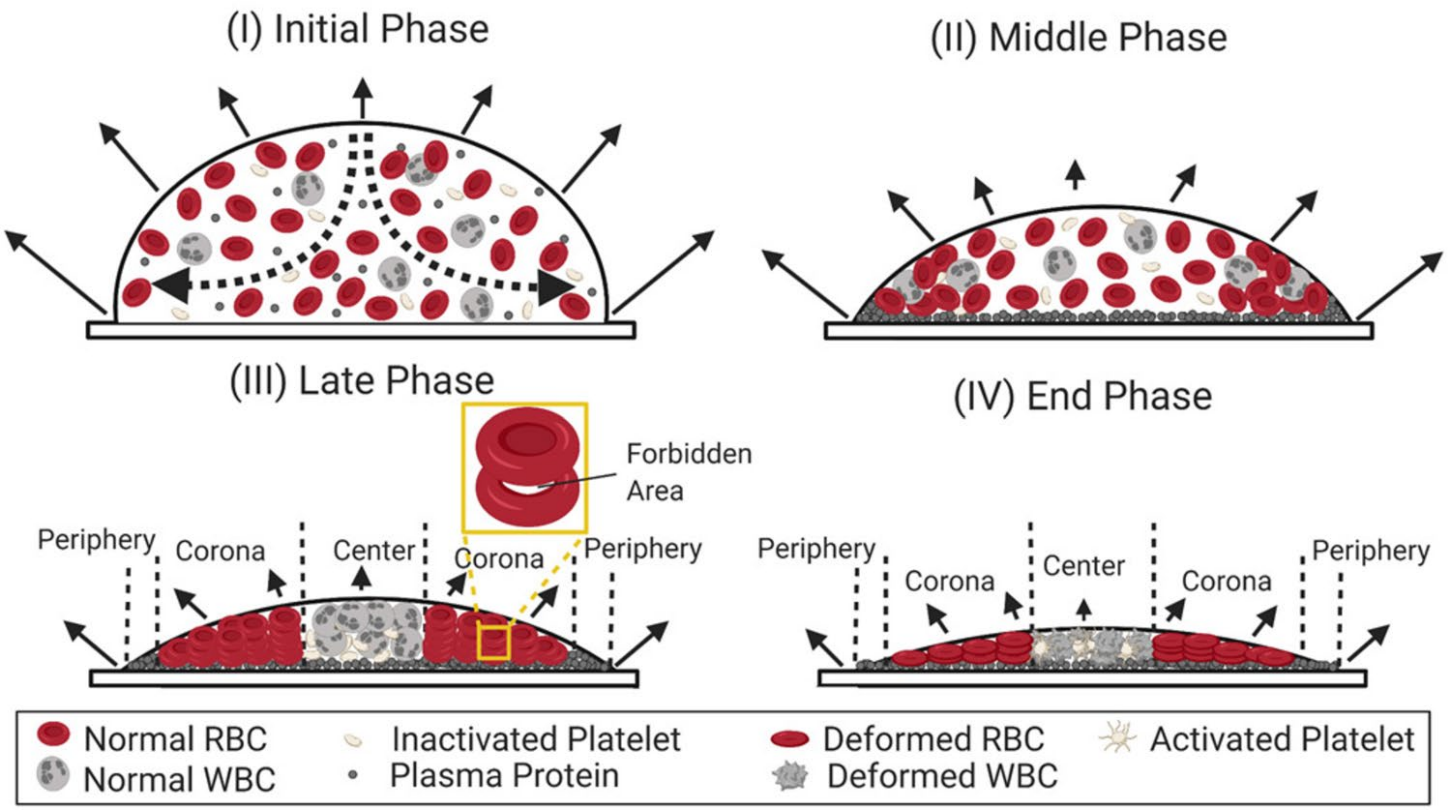
Side view of the self-assembling mechanism in the whole blood ($\phi$ = $100\%$) during the drying evolution: (I) The *initial phase* starts when the droplet is deposited on the substrate. The evaporation rate is highest near the three-phase contact line (depicted with solid arrows). The components (RBCs, WBCs, platelets, and proteins) are distributed randomly. A dotted line indicates that a capillary flow is developed in the droplet. (II) The *middle phase* begins when these components are carried towards the periphery. These components initiate interactions as soon as they come in contact with each other. Meanwhile, a plasma-rich layer is developed on the substrate. (III) The segregation of these components takes place as the water continually evaporates from the droplet. During the *late phase*, the three distinct regions (the periphery, the corona, and the center) are developed. The proteins are mostly present in the periphery. The WBCs and the platelets are pushed towards the central region. The RBCs are stacked in the corona region. The trapped water in between two RBCs is marked as the “forbidden area”. (IV) The deformation of the RBC and the WBC structures and the activation of the platelets take place during the *end phase*.

Figure 5(I–III)a shows the regions of interest at $\phi$ of 100, 75, and $12.5\%$ (v/v) respectively. The yellow, the orange, and the black squares display the zoomed view at the periphery and the corona, the interface of the corona and the central regions, and the central region, respectively. Fig. 5(I–III)b exhibits a smooth film in the corona region, and becomes more uniform with the increasing dilution of the blood sample. The cracks of the peripheral region at $\phi$ of 100 and $75\%$ (v/v) [Fig. 5(I–II)b] consist of some ellipsoidal structures which lead to inhomogeneity within the cracks, whereas no such peripheral region is found at $\phi$ of $12.5\%$ (v/v) (Fig. 5(III)a–b). At the interface of the corona and the central regions, some irregular troughs and ridges are revealed from the images of Fig. 5(I–II)c,d. A complex image is visible in Fig. 5(I–II)d, where the crack lines are not sharp and contain hair-like structures. which smoothly diminish in appearance in the diluted blood sample. Interestingly, these results show that the dilution at $\phi$ of $75\%$ (v/v) [Fig. 5(II)] doesn’t exhibit any significant changes in the distribution of these structures from the whole blood [Fig. 5(I)]. However, a marked difference can be seen at $12.5\%$ (v/v) [Fig. 5(III)]. Unlike other concentrations, some unique blunt spicules are present on the film (Fig. 5(III)b–c), and some aggregated sickle or oblate shaped structures are observed in the central region in Fig. 5(III)d.

## Discussion

### Microscopic structures of dried blood film

The SEM images in Fig. 5(I–III) allow us to determine the distribution of various components in the dried film. The components that can be distinguished from these images are plasma proteins, red blood cells (RBCs), white blood cells (WBCs), and platelets. The periphery of the film and the textures within the cracks indicate that the plasma proteins interact with the substrate and form a plasma-rich layer as soon as the droplet is deposited.

Most of these cracks in the peripheral region consist of fragmented or ellipsoidal RBCs (Fig. 5(I–II)b). None of the samples [$\phi$ of 100, 75, and $12.5\%$ (v/v)] show any blunt spicules (which resembles echinocyte, a deformed structural form of RBCs reported in^39^) in these cracks. This indicates that RBCs do not interact with the substrate directly; however, it does with the plasma-rich layer. Had these RBCs adsorbed on this substrate, their membranes would have different electrostatic interactions and might have led to the echinocytes, which are not (obviously) observed here. At the upper film surface, the corona region reveals smooth fragmented sheets. This suggests that the RBCs are stacked in a packed fashion such that their membranes form this sheet, a similar phenomenon reported in^40^. The compressed RBCs are raptured, as could be seen in the crack walls due to the mechanical stress curving the cracks (shown in Fig. 5(I–II)b).

Close observation of the film from the thin periphery region across the corona and into the central region indicates that the RBCs are predominantly in the corona. Any of these platelets and WBCs are not present in the periphery and in the corona. Moving from the corona to the central region, we see the concentration of the WBCs, the platelets and some residual RBCs. Fig. 5(I–II)c displays the irregular troughs and ridges which would resemble the different functional forms of the WBCs (microvilli structures reported in^41,42^) and some of the residual echinocytes. The crowdedness of the RBCs, the WBCs, and the plasma proteins is clearly illustrated by the fact that the dried film at $\phi$ of $100\%$ (v/v) [Fig. 5(I)a] contains ${\sim } 500 \times 10^{4}$ of RBCs, ${\sim } 1 \times 10^{4}$ of WBCs, and ${\sim } 40 \times 10^{4}$ of platelets, in addition to the initial salts and other molecular blood factors^14^. The crack lines are not sharp enough and contain hairy structures. These hairy structures are believed to be the activated form (a spreading structure with extended filopodia mentioned in^43^) of the platelets (Fig. 5(I–II)d). It confirms the existence of a complex combination of interactions among these components which can activate these platelets in response to the mechanical stress.

At $\phi$ of $12.5\%$ (v/v), the film only contains ${\sim } 60 \times 10^{4}$ of RBCs, ${\sim } 0.12 \times 10^{4}$ of WBCs, and ${\sim } 5 \times 10^{4}$ of platelets, a decrease of about a factor of 10. This significant reduction in their counts influences the morphology of the film, and is denoted in Fig. 5(III)a. The peripheral region does not appear in these images. Furthermore, the fragmented sheets in the corona region appear to be smoother, and its width gets reduced compared to other $\phi$. Some spicule-like structures are identified in the transitional region from the corona to the central regions (Fig. 5(III)b–c); and, some sickle and oblate-shaped structures are observed near the center (Fig. 5(III)d). The reduction (of RBCs, WBCs and platelets) concentrations decreases their interaction among themselves and with each other such that different functional forms of these components are observed. Moreover, as dilution increases, any interaction among the components within blood becomes negligible such that the inactivated form of these platelets are preserved, mostly observed in the central region.

### Concentration-driven phase transition in the drying droplets and the dried films

The outcome of three experimental observations support the presence of concentration-driven phase separation in the samples of a multi-colloid self-interacting solution, such as the whole blood. Our observations (drawn based on these three experimental outcomes) are independent of each other and facilitate measuring different aspects of the drying process including the final dried film morphology.

The first observation is related to the contact angle ($\theta$) measurements as a function of time during the drying process. The $\theta$ is found to decrease monotonically. Subsequently, a peak is observed for the whole blood. This pattern is observed in the dilution ranging from 100 to $62\%$ (v/v) [Figs. 2(I) and S1-S3(I)]. The peak, however, starts disappearing in the diluted samples starting from 50 to $12.5\%$ (v/v) [Figs. 2(II) and S1-S3(II)]. During this stage, $\theta$ exhibits a steep monotonic decrease. The third independent measurement is done on the morphology of the dried film. The mean width of the corona ($\overline{w}$), the mean crack spacing ($\overline{x}_c$) and the mean crack widening (${\overline{\lambda }}_c$) are found to be the highest for the whole blood [$\phi =100\%$ (v/v)]. All these parameters; however, are reduced as the initial concentration of the blood sample is diluted [Fig. 4(I–III)]. Interestingly, a smooth gradual decrease in these extracted parameters ($\overline{w}/ \overline{R}$, $\overline{x}_c$, and ${\overline{\lambda }}_c$) is not observed when varying with $\phi$. Had that been the case it could have assumed that the droplets experience a universal mechanism throughout the drying process, which is not the case here. Instead, a pronounced break in the trends of the parameters is seen at about $62\%$ (v/v). Comparing the R-squared values for the double linear fits with a single linear fit clearly favors the use of two linear fits—one from 100 to $62\%$ (v/v) samples, and, the other for 62 to $12.5\%$ (v/v). This behavior for the parameter $\overline{w}/ \overline{R}$ is found to be weakest, but still present, and is likely due to the fact that the width formation is the result of the deposition of the components during the convective flow, especially in the early stages of drying. Dilution of the whole blood (whether DI water or PBS) lowers the number of the components, and so reduces the corona’s width. Moreover, the formation of the corona is strongly influenced by the drying-driven fluid circulation, the droplet geometry, and its wetting. It is; however, not so dependent on the type of the components, but just the amount. In contrast, $\overline{x}_c$ and ${\overline{\lambda }}_c$ show a much stronger break and evidence for two linear regimes from 100 to $12.5\%$ (v/v).

All these three measurements indicate that there are two classes of the mechanisms involved. These mechanisms could be interpreted in the following way. A large number of components (such as RBCs, WBCs, platelets, proteins, etc.) are present in ${\sim } 2$ mm diameter droplet at $\phi = 100\%$ (v/v) [the whole blood]. These components are randomly distributed as soon as the droplet of the whole blood is deposited on the substrate [Fig. 6(I)]. In this *initial phase*, the evaporation rate is observed to be highest near the three-phase contact line [indicated with solid arrows in Fig. 6(I)]. A capillary flow [marked with dotted lines in Fig. 6(I)] is observed to develop and most of these components are carried towards the periphery. Subsequently, a plasma-rich layer is developed on the substrate. Soon after, all these components start moving closer to each other, which indicates the beginning of the *middle phase* in Fig. 6(II). In this phase, these components experience compression, or stretch, and shear at the same time near the periphery. The concentration of these components within the droplet increases as the water constantly evaporates. The confinement in the droplet diameter and the presence of the huge number of these components is likely to influence the activity of one component on others. Such interactions may result in a strong, complex combination of the potential chemicals amongst these various components. For example, a few of these components may try to arrange themselves in their original shape (elastic behavior); however, their arrangement would then create negative stress on other components. These other components may then rearrange (viscous behavior) themselves to accompany the accumulated stress. The elastic behavior can be modeled as $\sigma =E\epsilon$ where $\sigma$, *E*, and $\epsilon$ is the stress, averaged elastic modulus resulted from the arrangements of these components and the strain which occurs under the accumulated stress respectively. The visco-elastic behavior can be re-modeled by adding the Kelvin–Voigt element, i.e., $\sigma =E\epsilon +\eta {\dot{\epsilon }}$, where, $\eta$ is the viscosity, and ${\dot{\epsilon }}$ is the change of stain in respect to time *t*. This speculated viscous behavior is most likely governed by the interacting capabilities of the WBCs (with the other components viz., the RBCs and the platelets) owing to its largest size (${\sim } 15$ $\upmu$m) compared to the other components (the RBCs and the platelets are of ${\sim } 7$ $\upmu$m, and ${\sim } 2$ $\upmu$m respectively). The large peak that was noticed at $\phi$ of $100\%$ (v/v) [Figs. 2(I) and S1-S3(I), the presence of a similar peak has also been reported in^20^, may have some correlation to this viscous behavior during the drying process. However, the experiments reported in^20^ did not concentrate on diverse ranges of dilution; and hence the study failed to observe the transition at $62\%$. The current study, thus, contributes a crucial information related to phase transition. It would be interesting to conduct an in-situ rheology and mass measurements to directly probe these mechanisms; however, this is beyond the scope of the paper. The segregation of these components takes place during the *late phase* of the drying process [Fig. 6(III)]. The confinement of their sizes does not allow both the RBCs and the WBCs to be present within the periphery [evident from the SEM results shown in Fig. 5(I)a–b]. Most of the RBCs are organized in stacks in the corona region, and the WBCs and the platelets are pushed towards the central region [Fig. 6(III)]. There is a possibility that some of the water is trapped between two RBCs during the stacking of the RBCs, [marked as the “forbidden areas” in Fig. 6(III)]. The change of the texture from the light to dark gray texture (Fig. 1(I)c–d) might be related to the final evaporation of this entrapped water. The loss of water in these droplets during the drying process creates a hypotonic environment to the cellular components present in the blood. The blood cells are more likely to undergo hemolysis inducing a fundamental change in their composition. Furthermore, the developing mechanical stress evolved due to the loss of the water from the droplet deforms the structures of RBCs and the WBCs in the *end phase* [Fig. 6(IV)]. The inactive platelets are activated in response to the mechanical stress and are observed as the hairy structures along the crack lines (indicated in Fig. 5(I)d).

As mentioned already, the whole blood sample is diluted by adding different volumes of de-ionized water. Obviously, the initial concentration of these components in the diluted blood samples [$\phi$ ranging from 85 to $12.5\%$ (v/v)] becomes less compared to the whole blood. The addition of de-ionized water rather than PBS (the buffered saline) is likely to trigger a hypotonic environment to these cellular components prior to the drying process. However, it is to be noted that the changes (if any) occur in the initial mixtures before deposition but may be balanced as the water evaporates. Exploring this effect would be interesting but is beyond the scope of this paper. Additional measurements were carried out in a parallel way but diluting with PBS, in order to maintain the native environment of the cells and the fundamental changes (hemolysis) in the blood composition (from intact to fragments) could be avoided. These results appear consistent with the DI water dilution and so minimizes this mechanism. However, it is worth mentioning that the dilution of the blood by PBS (at its fixed concentration) will increase the appearance of salts residues or crystals^20^, which influences the statistics derived from the optical images, and lead to artifacts in the scanning electron microscopy images.

The drying process of the diluted blood samples exhibits similar phases [from *initial* to the *end phase* shown in Fig. 6(I–IV)]. Figure 1(I–II)a indicates a change in the texture (from dark to light gray) as soon as the first image during the drying process is captured. The normalized plot in Fig S4(I-II)a shown in the supplementary information directly illustrates this change in the texture. At $\phi$ from 100 to $62\%$ (v/v), the mean intensity value of the first image is ${\sim } 20\%$ of the total value, further dilutions of the concentration leads the mean intensity to be at ${\sim } 80\%$ of the total value. It is to be noted that RBCs contain hemoglobin protein (which is responsible for the redness). The change of texture, or the rise in the normalized plot, indicates that the number of RBCs reduces with the diluted concentrations. The decrease of RBCs also ensures that the other components will also minimize to maintain their relative initial concentrations. The number of these components gradually decreases with the diluted concentrations [in $\phi$ ranging from 85 to $62\%$ (v/v)] and leads to a reduction in the chemical potential amongst them. The shrinkage and disappearance of peak in the contact angle measurements supports this mechanism. In addition, the observed shift in the peak to the later time (depicted by green rectangles in Fig. 2(I)a–d) indicates that these diluted samples require more time (greater concentration and assembly) to reach the visco-elastic tipping point of the system. Furthermore, the wetting angle peak at the concentration range of 100 to $62\%$ (v/v) is found to be present in spite of varying the physiological and environmental conditions. The blood diluted with PBS [see Fig. S1(I)], and the new blood batch deposited on different substrates [see Figs. S2-S3(I)] have been examined. We noticed a similar peak in the contact angle measurements. Nonetheless, the nature of the peaks varies, i.e., in some cases, the peak is delayed, while in some other cases, the peak is broadened or reduced, depending on the wettability of these droplets. This systematic study of diluted samples (ranging between 100 and $12.5\%$ (v/v) that include all the possible variations in terms of physiology and environments firmly establish a very important point, i.e., the observed peak is not specific to the blood (samples) diluted with de-ionized water only; rather it confirms that the peak is a trademark that establishes a general phenomenon of the concentration-driven self-assembly.

The presence of WBCs also seems to act as a mediator which favors the interactions between various components, rather than the interaction between these components and the substrate. This process results in sliding of the cracked domains over the attachment of these domains with the substrate in the samples ranging from 100 to $62\%$ (v/v). Furthermore, this process shows a sharp decrease in $\overline{x}_c$ and ${\overline{\lambda }}_c$. In contrast, for $\phi$ from 50 to $12.5\%$ (v/v), the number of these various components is reduced enough that the chemical potential related to WBCs becomes negligible. This results in the switching of the mechanism from the visco-elastic to elastic behavior. No peak observed at this concentration range in the contact angle measurements [Figs. 2(II) and S1-S3(II)]. Moreover, the attachment of the film to the substrate becomes stronger, the sliding of the crack domains stops, and more radial cracks appear on the film surface [Fig. 1(II)].

Our findings, thus, clearly establish the existence of a sharp phase transition in the whole blood through a simple physical drying process. This transition relied on the concentration of the various components present in the droplet. It reveals essential information about the self-assembling mechanism in a multi-component bio-colloid, such as the whole human blood. Our systematic study of the dilution range also ensures that the concentration-driven phase transition is not specific to the blood-water system only; rather it is a general phenomenon of drying-driven diluting blood droplets. Finally, this study demands theoretical attention for such a multifaceted phase transition that relies on the complex combination of interacting chemical potentials for unearthing the hierarchical structures present in nature.

## Methods

### Materials and sample preparation

A 1 $\upmu$L of whole blood contains 400 to $500 \times 10^{4}$ of RBCs, 0.5 to $1 \times 10^{4}$ of WBCs, 14 to $40 \times 10^{4}$ of platelets, depending on the pathological condition of a donor, along with a small amount of plasma proteins (fibrinogen, immunoglobulins, albumin) and salt ions^14^. The typical size of RBCs, WBCs, and platelets are 6 to 8 μm, ${\sim } 15$ $\upmu$m, and 2 to 3 $\upmu$m respectively^14^. The RBCs are red in color due to the presence of the iron-containing protein, hemoglobin. Furthermore, RBCs contain a membrane as well. A membrane is made of $19.5\%$ water, $39.6\%$ proteins, $35.1\%$ lipids and $5.8\%$ (w/w) carbohydrates^44^. These cellular components in the blood can alter their shapes in response to the toxicity or pH or shear stress^43^.

For example, the healthy RBCs are of bi-concave or discoid shape; however, these transform into different forms such as ellipsoidal, echinocyte (a round shape with short blunt spicules), sickle (crescent moon), teardrop, etc^44^. The WBCs, on the other hand, are white in color, and generally contain irregular and nucleated structure. These structures are capable of transforming themselves into different functional forms, irregular troughs and ridges (microvilli structures)^41,42^. The platelet, when inactivated, follows a discoid and an anuclear structure, but, changes into its spread form with extended filopodia on its activation. The cytoskeleton of platelets is composed of actin and actin-binding proteins; these can polymerize and activate those platelets in response to the environmental or chemical signals^43^.

The whole human blood used in this study was purchased from Lampire Biological Laboratories, USA^45^ and mixed with Na-Citrate anticoagulant (Catalog number 7203706). These samples are used in our experiments without any further chemical processing. except for dilution with DI water or PBS. The anticoagulants inhibit most of the platelets to aggregate, and prevent the coagulation of the blood.

Different concentrations ($\phi$) of blood were prepared from 85 to $12.5\%$ (v/v) by adding de-ionized water (Millipore, 18.2 M$\Omega$ cm at ~ 25 °C). A volume of ${\sim } 1$ $\upmu$L of sample was pipetted on a fresh microscopic coverslip (Catalog number 48366-045, VWR, USA) to form a circular droplet of radius ${\sim } 1$ mm at the room temperature of ${\sim } 25^{\circ }$C, and relative humidity of ${\sim } 50$ %. This procedure ensured that every droplet is precisely exposed to the same substrate conditions, and environmental conditions, and helped achieve uniform reproducibility of 3–4 repetitions. The time-point when the droplets are deposited on the coverslip is marked as the start-time of image acquisition. It is to be noted that the samples are prepared immediately before imaging.

A few experiments were carried out separately by varying environmental and physiological conditions to ensure the wettability of these droplets. One such experiment includes the concentration of the whole blood ($100\%$ by volume) from the same batch that is diluted by adding 1$\times$ phosphate buffer saline (PBS, BP243820, Fisher BioReagents, USA). 1$\times$ PBS has a concentration of 0.137M NaCl, 0.0027M KCl, and 0.119M phosphates that maintains a pH of 7.3–7.5. Similarly, different concentrations of diluted blood samples using PBS ($\phi _p$) were prepared that include the range of 75 to $12.5\%$ (by volume). A new blood batch from the same company (Catalog number 7203706, Lampire Biological Laboratories, USA) was ordered and exactly the same procedure was followed to dilute the blood by adding de-ionized water. The wettability of these diluted blood droplets is measured under two substrate conditions. The initial contact angle of these droplets is found to be ${\sim } 55^{\circ }$ and ${\sim } 85^{\circ }$ when deposited on various coverslips, Catalogs no. 48366-045, VWR, USA and 4867-100, R & D Systems, USA, respectively.

### Image acquisition

The time-lapse images are captured at every two seconds in bright field microscopy (Amscope, USA) using a $5\times$ objective lens. An 8-bit digital camera (MU300, Amscope, USA) attached to the microscope captured images at a fixed resolution of $3664\times 2748$ pixels. The pixels to real-space length scale was changed by using a calibration slide. The lamp intensity was kept fixed throughout the experiment to minimize the fluctuations in the background (coverslip) intensity. All the images were converted to the gray shade for clear visualization.

The contact angle of the prepared samples was measured during the drying process by the contact angle goniometer (Model 90, Ramé-hart Instrument Company, USA) to check the wettability of these droplets. Each experiment was repeated twice to ensure the reproducibility.

The microstructural analysis of the dried films was performed using scanning electron microscopy (JEOL-7000F, JEOL Inc. MA, USA). Prior to this, the dried films were sputter-coated with a ${\sim } 4$ nm thick layer of gold nanoparticles using EMS sputter coater to improve the quality of the sample surface’s conductivity. The SEM analysis was conducted using secondary electron at an accelerating voltage of 3 kV and probe current of 5 mA.

### Image analysis

Different parameters of the images were measured using ImageJ^46^. A circular region of interest (ROI) of the droplet image was selected by an *oval tool* in ImageJ. The mean and the standard deviation parameters of the first-order statistics (FOS) was extracted. No pre-processing of the images was required since the blood samples contain a high contrast difference compared to the background (coverslip). Furthermore, a 2-D plot profile was computed by selecting a rectangle along the horizontal diameter of the droplet, $\rho$. The width of the corona region was measured five times to compute the averaged width ($\overline{w}$). It was normalized by dividing it with the averaged radius ($\overline{R}$). The parameter, $\overline{w}$/$\overline{R}$ is then plotted at a function of $\phi$. To estimate the crack width ($\lambda _c$) and the crack spacing ($x_c$), a circular line was drawn, and their averaged values were also determined at each $\phi$. The detailed procedure of $x_c$ measurements can be found in^47^.

## Supplementary information

Supplementary information.

Supplementary Video 1.

Supplementary Video 2.

Supplementary Video 3.

Supplementary Video 4.

Supplementary Video 5.

Supplementary Video 6.

Supplementary Video 7.
