# Supplementary material for: Concentration-driven phase transition and self-assembly in drying droplets of diluting whole blood
Source: Sci Rep. 2020 Nov 3;10:18908. doi: 10.1038/s41598-020-76082-6 (PMC7609771; doi:10.1038/s41598-020-76082-6)
Supplement: Supplementary file 1 — Supplementary information. [file 41598_2020_76082_MOESM1_ESM.pdf]

Concentration-driven phase transition and self-assembly in drying droplets of diluting whole blood

Anusuya Pal[a †], Amalesh Gope[b], John D. Obayemi[c,d], and Germano S. Iannacchione[a*]

[a] *Order-Disorder Phenomena Laboratory, Department of Physics, Worcester Polytechnic Institute, Worcester, MA, 01609, USA*

[b] *Tezpur University, Tezpur, Assam, 784028, India*

[c] *Department of Mechanical Engineering,* [d] *Department of Bioengineering, Worcester Polytechnic Institute, Worcester, MA, 01609, USA*

[*] gsiannac@wpi.edu, [†]apal@wpi.edu

SUPPLEMENTARY INFORMATION

I. CONTACT ANGLE MEASUREMENTS OF THE DRYING BLOOD DROPLETS

A series of contact angle experiments were conducted to understand the complexity of different physiological environments and substrate variability that may affect the concentration-driven phase transition in these drying diluted blood droplets. To examine the effect of physiological environments, the diluent (i.e., the de-ionized water) was substituted to phosphate saline buffer (PBS). Furthermore, experiments were also conducted with different batch of blood those were examined using different substrates.

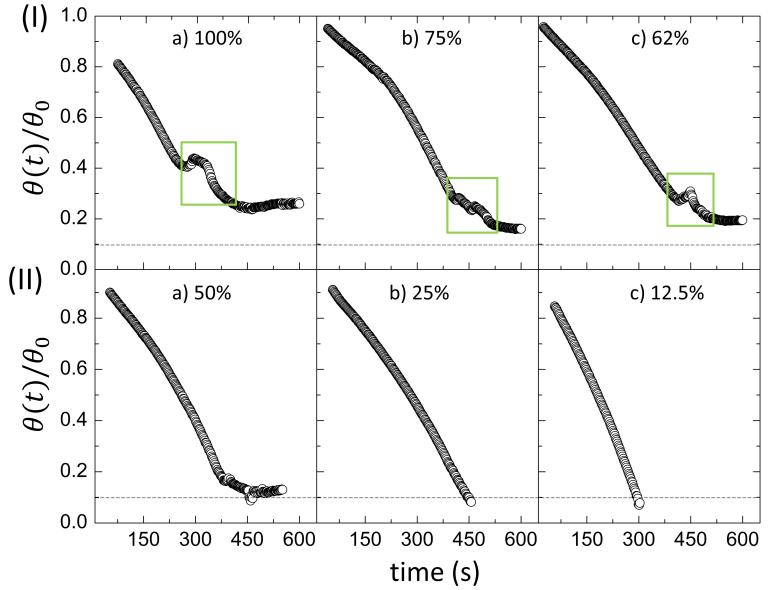

FIG. S1. Variations of the normalized contact angle ($\theta(t)/\theta_0$) during the drying process of the blood droplets at different ϕ_p ranging (I) from 100 to 62% (v/v), and (II) 50 to 12.5% (v/v) are shown. The normalization of $\theta(t)$ is obtained by dividing it with the angle at $t = 0$ (θ_0). A monotonic decrease in the contact angle measurements is associated with the presence of a peak-like feature (indicated with a green rectangle) is observed in (I). The disappearance of this feature in these measurements is depicted in (II).

Figure S1(I-II) shows the drying evolution of the normalized contact angle of the blood samples diluted by adding PBS at a fixed concentration of 1x. The diluting concentration (ϕ_p) is varied from 100 to 12.5% (v/v). The initial contact angle, i.e., the angle at $t = 0$

(θ_0) is found to be ∼55° for these sample sets. The blood batch is the same one that is used to portray the evolution in Fig. 2(I-II) in the (main) article. The peaks are observed in the diluted blood samples containing salts ranging from 100 to 62% (v/v). This indicates that the presence of the peak is a general trademark that is attributed to the self-assembling mechanisms of the constituent particles present in the droplet (and it is not limited to the blood-DI system only).

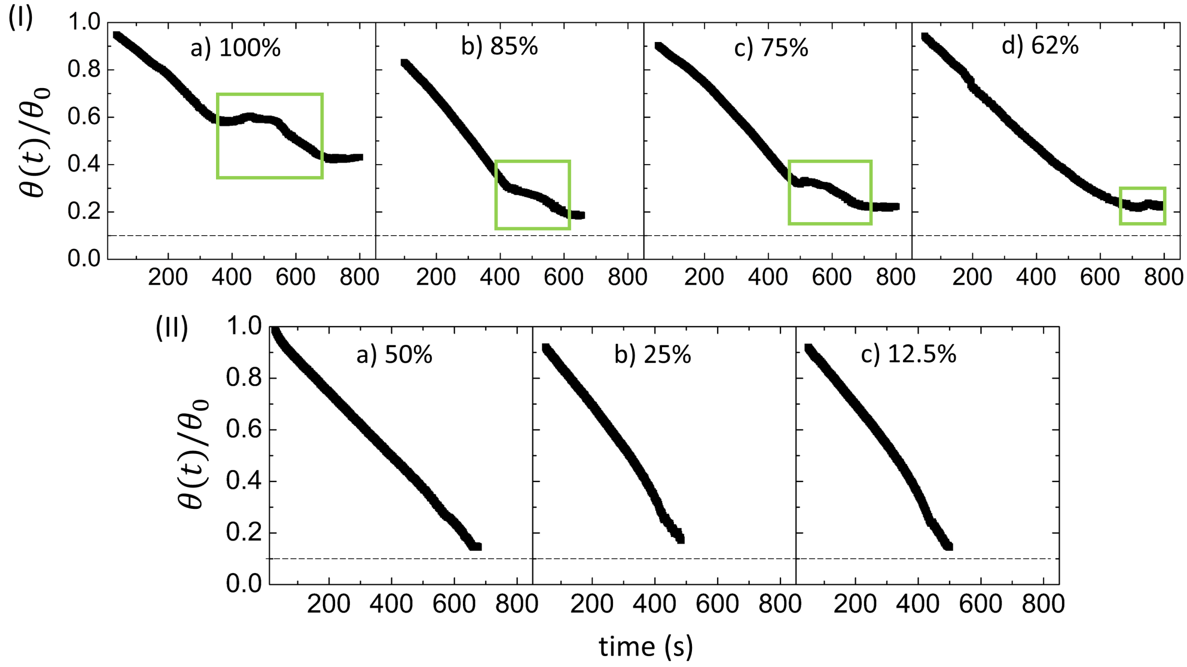

FIG. S2. Variations of the normalized contact angle ($\theta(t)/\theta_0$) during the drying process of the blood droplets at different ϕ ranging (I) from 100 to 62% (v/v), and (II) 50 to 12.5% (v/v) are shown. The normalization of $\theta(t)$ is obtained by dividing it with the angle at $t = 0$ (θ_0). A monotonic decrease in the contact angle measurements is associated with the presence of a peak-like feature (indicated with a green rectangle) is observed in (I). The disappearance of this feature in these measurements is depicted in (II).

Figure S2(I-II) shows the drying evolution of the normalized contact angle of the new batch of blood samples that is diluted by adding de-ionized water. The diluting concentration (ϕ) is varied from 100 to 12.5% (v/v). The initial contact angle, i.e., the angle at $t = 0$ (θ_0) is found to be ∼55° for these sample sets. The peaks also appear for the range of 100 to 62% (v/v) in the new batch of blood; however, the nature of the peaks seem to be

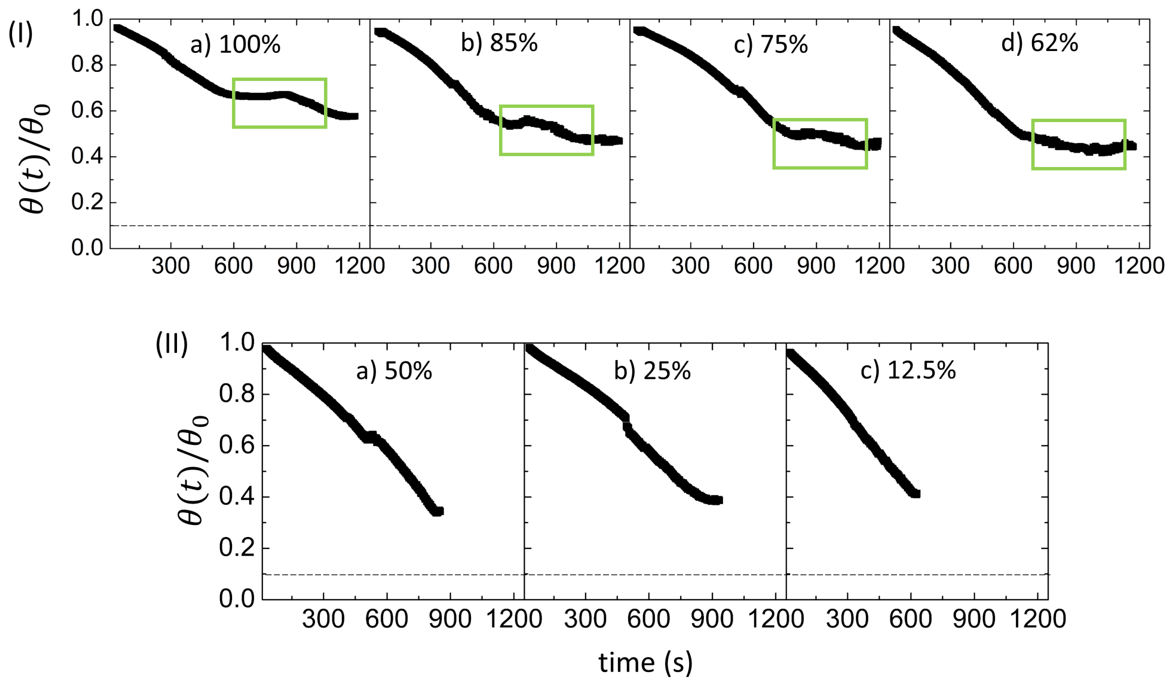

FIG. S3. Variations of the normalized contact angle ($\theta(t)/\theta_0$) during the drying process of the blood droplets at different ϕ ranging (I) from 100 to 62% (v/v), and (II) 50 to 12.5% (v/v) are shown. The normalization of $\theta(t)$ is obtained by dividing it with the angle at $t = 0$ (θ_0). A monotonic decrease in the contact angle measurements is associated with the presence of a peak-like feature (indicated with a green rectangle) is observed in (I). The disappearance of this feature in these measurements is depicted in (II).

different in two batches of the blood (see Fig. 2(I-II) in the paper). The differences in the peaks occurred in spite of the fact that these experiments are performed under the same conditions (droplet size, temperature, humidity, etc.). The apparent reason for the observed differences, however, is likely due to the diverse counts of cellular components, such as RBCs, WBCs, and platelets, present in all the healthy persons (the count is expected to be different from one person to another). The characteristics of these peaks, thus appear to be different since it is dependent on their counts, which may vary within the normal range from one healthy person to another.

Similar to Fig. S2(I-II), Fig. S3(I-II) also shows the drying evolution of the normalized contact angle of the same batch of blood samples diluted by adding de-ionized water; but on different substrate (coverslip). The initial contact angle, i.e., the angle at $t = 0$ (θ_0)

of the droplets for these coverslips is found to be ∼85°. A longer duration is noticed for the evolution of contact angle during the drying process (∼350 s more than the other ones) due to the larger initial contact angle irrespective of the similar environmental conditions (droplet size, volume, temperature, humidity, etc.). Furthermore, the peaks are broader compared to the rest [see Fig. S1-S2(I-II)]. The broadening and the delaying of this peak indicate that these diluted samples at greater initial contact angle take more time to reach a critical concentration at which the visco-elasticity of the cellular components can build up.

II. PLOT PROFILE OF THE DRYING BLOOD DROPLETS

The change in the textures of the images [shown in Fig. 1(I-II)] is directly mapped with the normalized plot in Fig. S4(I-II). A rectangle (containing a width of ∼0.6 mm) along the diameter was drawn on the images in such a way that a small portion covers the background (coverslip) on both sides to complete the normalization process. In this plot, the x-axis illustrates the distance along the horizontal diameter (ρ) of the droplet, and the y-axis displays the vertically normalized averaged pixel intensity. For the first captured image at ϕ varying from 100 to 62% (v/v), only the 20% of the total intensity value in the plot profile (Fig. S4(I)a) indicates the uniformity of the dark texture (Fig. 1(I)a). The texture of these images turns lighter gray (Fig. 1(II)a) with the increasing dilution [from 50 to 12.5% (v/v)]. It is evident if we notice the plot profile which shows the intensity as $60 - 80\%$ of the total value (Fig. S4(II)a). The texture of the droplet changes to gray as soon as the front moves from the periphery towards the center (Fig. 1(I-II)b). It is indicated by the increasing normalized intensity near the edge of the droplet (Fig. S4(I-II)b). In the next phase, the gray texture appears in the central region of the droplet shown in Fig. 1(I-II)b. This appearance is predominantly marked with a broad hump-like feature at the center of the plot profile from 100 to 75% (v/v). However, this feature starts disappearing from 62 to 12.5% (v/v) [Fig. S4(I-II)b]. The next stage is identified with the propagation of the radial cracks and their widening and branching (Fig. 1(I-II)c-d). The plot profile in Fig. S4(I-II)c-d shows the way the depth of the cracks decreases with the (increased) dilution by tracking the spikes from 100 to 12.5% (v/v). This observation indicates the thinning of the dried film and the decrease in the depth of their intervening cracks.

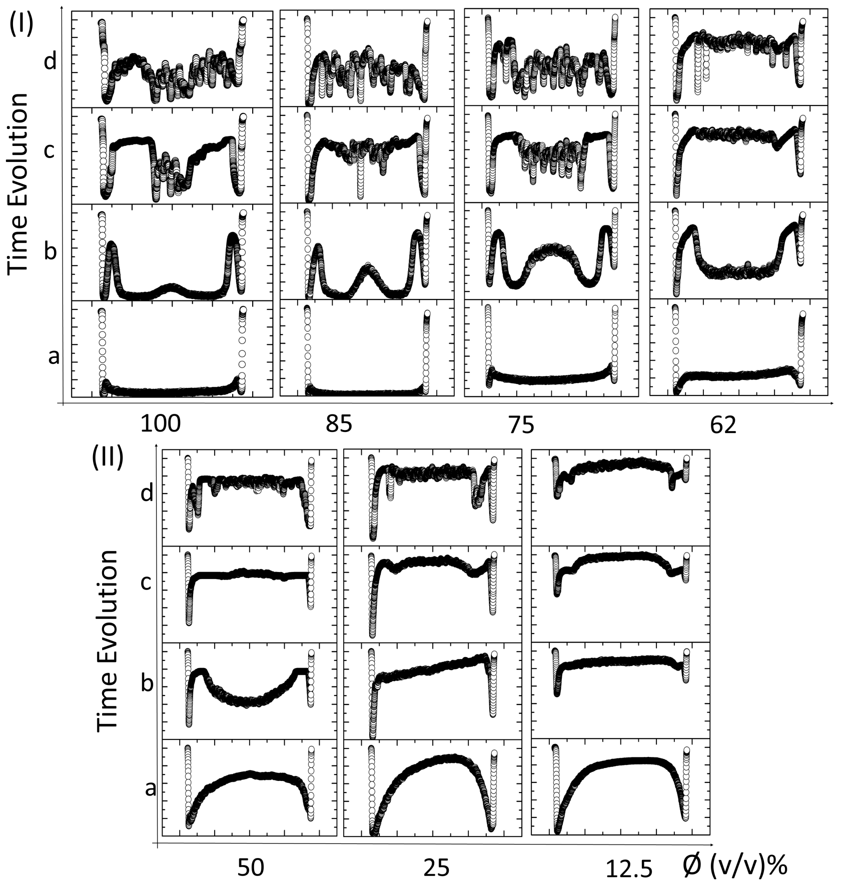

FIG. S4. A normalized intensity of a 2-D plot profile along the horizontal diameter of the droplet (ρ) is shown for corresponding a-d depicted in Fig. 1(I-II). These plots captured during the drying process at each ϕ: (I) from 100 to 62% (v/v), and (II) from 50 to 12.5% (v/v). The intensity of the captured image rises along ρ when the dilution is increased (I-II)a. The movement of the fluid front from the periphery is illustrated by a hump in both the edges of the profile (I-II)b. An appearance of the hump in the central region of the profile is observed in (I)b, whereas its disappearance is evident in (II)b. The propagation of the cracks disturbs the uniformity of the plot profile from 100 to 62% (v/v) in (I)c-d; however, it is hardly visible in (II)c-d. The x ranges from -1.25 to 1.25 mm (with a step size of 0.5 mm), while the y axis varies from 0 to 1.1 arbitrary units (a.u.) (with step size of 0.2 a.u.).

III. TEXTURAL IMAGE ANALYSIS OF THE DRYING BLOOD DROPLETS

The mean values at various ϕ depicts a slow increase in the initial drying phase. Subsequently, a rapid rise to a maximum value [Fig. 3(I-II)] is also observed. It reduces in

the later stage of the drying process and saturates towards the end. The mean increases to ∼35 a.u. [from ∼15 arbitrary units (a.u.)] at ϕ of 100 to 62% (v/v) [Fig. 3(I)a-d]. However, it initiates from $50 - 70$ a.u. and expands by $10 - 15$ a.u. at ϕ of 50 to 12.5% (v/v) [Fig. 3(II)a-c]. This phase lasts for $400 - 500$ seconds for the ϕ varying from 100 to 62% (v/v) and for $550 - 650$ seconds from 50 to 12.5% (v/v). Across the x axis, the rapid rise in the mean values is observed, which is identified as the next phase. This phase can clearly be distinguished for 100 to 62% (v/v); however, it is not the case for the rest. The phase becomes short, and the area covered by the peak starts decreasing with the increase of the dilution. The saturated value of the mean is smaller than the maximum value for ϕ of 100 to 75% (v/v) [Fig. 3(I)a-c], whereas, from 62 to 12.5% (v/v), these values are nearly close. A comparison of the time-lapse images [Fig. 3(I-II)] with the statistical image analysis [Fig. 3(I-II)] confirms that the initial increase in the mean values is up to the time when the fluid front moves uniformly from the periphery to the central region. At this stage, the appearance of the gray texture arises from the periphery, and it is displayed with the slow increase of the mean intensity. The moment this gray texture starts developing from the central region of the droplet, the mean exhibits a rapid rise (Fig. 1(I-II)b). It is to be noted that the evolution of the peak-like feature in the contact angle measurements (illustrated by the dashed lines in Fig. 2(I)a-d) occurs during this phase where the mean shows the slow and the slight rapid increase. As time progresses, the cracks appear and mark the next stage of the drying process (Fig. 1(I-II)c-d). The branching and the widening of the radial cracks alter the gray texture from light to dark from 100 to 75% (v/v). A reduction of ∼20% is observed when we compared the saturated values to the maximum values. However, these values are found to be nearly close from 62 to 12.5% (v/v) [Fig. 3(I-II)].

In addition to the mean, another extracted FOS parameter is the standard deviation (SD). The SD is found to be more sensitive to the local changes in the images than the mean. The SD rises initially for ϕ from 100 to 75% (v/v) in Fig. 4(I)a-c, and a first peak (depicted with a red-colored rectangle) is observed. The mapping of SD with the mean confirms that this peak emerges exactly at the same time at which the mean starts the rapid rise phase. Subsequently, the peak reduces and forms a dip around the time at which the mean shows its peak behavior. In the later stage, the SD increases to establish a second peak (displayed with a red-colored circle). It starts broadening from ϕ of 85% (v/v), and diminishes at $\phi = 62\%$ (v/v). A second dip begins in the initial drying phase of the same

ϕ [62% (v/v)] (Fig. 4(I)d highlighted with a red-colored star). This second dip gets wider (Fig. 4(II)a) at ϕ of 50% (v/v). A further increase of ϕ allow the second dip to engulf the first one and. A very different behavior is observed at ϕ of 25 and 12.5% (v/v), respectively. The SD is reduced to a minimum, and rises to a maximum and finally saturates at ϕ of 25% (v/v) in Fig. 4(II)b. In contrast, at ϕ of 12.5% (v/v) in Fig. 4(II)c, the SD decreases just the way it does at ϕ of 25% (v/v); however, it (SD) hardly increases at the end of the drying process. A comparison of the time-lapse images (Fig. 1(I-II)a-d) with the statistical image analysis [Fig. 4(I-II)] shows that the initial slow increase in the SD values is up to the time when the fluid front or the gray texture appears uniformly from the periphery to the central region [similar to the mean from 100 to 75% (v/v)]. The evolution of the peak-like feature in the contact angle measurements (illustrated with dashed lines in Fig. 4(I)a-d) emerges during this phase where the SD increases to reach the maximum values. The moment the maximum gray texture appears in the central region of the droplet (Fig. 1(I-II)b), the mean reaches a peak, whereas the SD shows a dip at ϕ of 100 to 75% (v/v). In contrast, a decrease in the SD values during the initial stage of the drying at ϕ of 62% (v/v) is predominantly observed; and it continues to decrease for the rest of the ϕ from 50 to 12.5% (v/v). The SD increases to the second peak and then decreases during the stage of the crack propagation. The decrease of the SD values from this second peak is predominantly observed during the time at which the gray texture of the domains created by the radial cracks changes from light to dark gray. This fact is evident from 100 to 75% (v/v), and we see a reduction of the SD by ∼10% when it is compared to the saturated values to the second peak values. On the other hand, at ϕ of 62 to 12.5% (v/v), these values are found to be almost equal [Fig. 4(I-II)]. Thus, the FOS parameters follow the image textural changes during the drying process, providing consistent statistical results to differentiate the stages and an evidence for the phase transition at 62% (v/v) during the drying process at all ϕ.

IV. LEGENDS OF ALL THE SUPPLEMENTARY VIDEOS PROVIDED

All the diluting concentrations (ϕ) are prepared by adding different volumes of de-ionized water with the whole human blood.

1. `V1_100%` is the video that shows the drying evolution of the whole human blood at ϕ of 100%.

2. V2_85% is the video that shows the drying evolution of the whole human blood at ϕ of 85%.

3. V3_75% is the video that shows the drying evolution of the whole human blood at ϕ of 75%.

4. V4_62% is the video that shows the drying evolution of the whole human blood at ϕ of 62%.

5. V5_50% is the video that shows the drying evolution of the whole human blood at ϕ of 50%.

6. V6_25% is the video that shows the drying evolution of the whole human blood at ϕ of 25%.

7. V7_12.5% is the video that shows the drying evolution of the whole human blood at ϕ of 12.5%.
